# Descriptions of two new species of *Aelurillus* Simon, 1884 (Araneae, Salticidae) from the Mediterranean, with the synonymization of *A. steliosi* Dobroruka, 2002

**DOI:** 10.3897/zookeys.516.9439

**Published:** 2015-08-10

**Authors:** Galina N. Azarkina, Marjan Komnenov

**Affiliations:** 1 The Laboratory of Systematics of Invertebrate Animals, The Institute of Systematics and Ecology of Animals, SB RAS, Frunze Street 11, Novosibirsk 630091, Russia; 2 Department of Molecular Biology and Genetics, Democritus University of Thrace, 68100 Alexandroupoli, Greece

**Keywords:** Aranei, jumping spiders, Mediterranean, synonymy

## Abstract

Two *Aelurillus* species are described as new, *Aelurillus alboclypeus* **sp. n.** (♂♀, from Turkey) and *Aelurillus deltshevi* **sp. n.** (♂, from Macedonia, Bulgaria and Azerbaijan). *Aelurillus steliosi* Dobroruka, 2002 is synonymized with *Aelurillus leipoldae* (Metzner, 1999). Additional distributions of the closely related species *Aelurillus v-insignitus* are provided for the region of study. Distributional maps are provided for the five species reported in this paper.

## Introduction

To date, 69 species and two subspecies of *Aelurillus* have been described in the world fauna (World Spider Catalog 2015). The genus *Aelurillus* is distributed predominantly in the Palaearctic Region, with only ten species being recorded from outside its limits. The fauna of the Balkans, Turkey and Azerbaijan consists of 14 species of *Aelurillus*. The best studied region is Greece containing ten species (Deltshev and Paraschi 1990; Metzner 1999; Azarkina 2002; Dobroruka 2002; Logunov and Chatzaki 2003; Bosmans and Chatzaki 2005; Azarkina and Logunov 2006; Bosmans et al. 2009; Russell-Smith et al. 2011). Four species have been recorded from Macedonia (Komnenov 2002; 2003; 2006; Fišer and Azarkina 2005), four species from Turkey (Topçu et al. 2005; Danişman et al. 2012; Azarkina and Mirshamsi 2014, Coşar et al. 2014, Logunov 2015), two from Azerbaijan (Logunov and Guseinov 2002) and one from Bulgaria (Deltshev et al. 2001, Lazarov 2005). Four of the 14 species recorded from the region at hand are regional endemics: two species from Crete (*Aelurillus cretensis* Azarkina, 2002 and *Aelurillus leipoldae* (Metzner, 1999)) and two species from Paros Island, Greece (*Aelurillus guecki* Metzner, 1999 and *Aelurillus steinmetzi* Metzner, 1999).

Logunov and Chatzaki (2003: 96) proposed *Aelurillus steliosi* as synonym of *Aelurillus cretensis* “It is safe to assume that this species is a synonym of *Aelurillus cretensis*”. Bosmans and Chatzaki 2005 mentioned *Aelurillus steliosi* in reference to *Aelurillus cretensis*, but synonymization was confirmed only in Bosmans et al. 2013 followed by Logunov and Chatzaki 2003. However this synonymization was provided without examination of any type material. In this paper the correct synonymization of *Aelurillus steliosi* is established, with *Aelurillus leipoldae* based on type material. Two new species are also described, *Aelurillus deltshevi* sp. n. (♂, from Macedonia, Bulgaria and Azerbaijan) and *Aelurillus alboclypeus* sp. n. (♂♀, from Turkey), and a new synonymy of *Aelurillus steliosi* Dobroruka, 2002 with *Aelurillus leipoldae* (Metzner, 1999) is proposed to replace an erroneous one (Bosmans et al. 2013).

## Material and methods

This paper is based on both museum collections and newly collected material from Macedonia, Bulgaria, Greece and Turkey. Specimens were studied in ethanol and their colours refer to those of the preserved specimens. All drawings were made with the aid of a reticular eyepiece attached to an MBS-10 stereomicroscope. The male pedipalps and epigynes were detached for study. Epigynes were macerated in 20% KOH solution for one night. After being drawn, the copulatory organs were placed in microvials or small pieces of paper with ethanol together with the specimens from which they had been removed. Digital images were taken with a Zeiss Stemi 2000 and an attached Canon EOS 550D camera. Stack images were combined using Helicon Focus software. All drawings were edited and assembled in Adobe Photoshop. Distribution maps were produced using the online mapping software SimpleMappr (Shorthouse 2010) with minor modification.

Specimens for this study were borrowed from or placed in the following museums and personal collections:

ISEA Institute for Systematics and Ecology of Animals, Novosibirsk, Russia (G. N. Azarkina);

IZSB Institute of Zoology, Sofia, Bulgaria (C. Deltshev);

LM World Museums Liverpool, Liverpool, UK (G. Night);

MMUM Manchester Museum, University of Manchester, Manchester, UK (D.V. Logunov);

MNHN Muséum national d’Histoire naturelle, Paris, France (E.-A. Leguin);

NHM Natural History Museum, Vienna, Austria (J. Gruber);

NHMC Natural History Museum, University of Crete, Crete, Greece (A. Trichas);

PCHM Personal collection of H. Metzner (Burghaslach, Germany);

PCMK Personal collection of M. Komnenov (Scopje, Macedonia);

SMNK State Museum of Natural History, Karlsruhe (H.Höfer);

SNHM Senckenberg Natural History Museum, Frankfurt am Main, Germany (P. Jäger).

Abbreviations used in the text: AME – anterior median eyes, ALE – anterior lateral eyes, PLE – posterior lateral eyes, Fm – femur, Pt – patella, TA – terminal apophysis; Tb – tibia, Mt – metatarsus. The sequence of leg segments in measurement data is as follows: femur+patella+tibia+metatarsus+tarsus. All measurements are in mm. For the leg spination the system adopted is that used by Ono (1988).

## Taxonomy

### Aelurillus alboclypeus sp. n.

Taxon classificationAnimaliaAraneaeSalticidae

http://zoobank.org/F680F462-FC96-4652-AE0E-265C11C1B249

Figs 1 , 2–13 , 14–15

Aelurillus gershomi : Danişman et al. 2012: 215 (misidentification); Coşar et al. 2014: 84 (misidentification).

#### Type material.

**Holotype**: ♂ (ISEA 000.287) TURKEY, Antalya Province, 18 km SSE of Elmali, Bey Mt. Range, 6 km WSW of Kızlarsivrisi Mt., 1800–2000 m a.s.l., 36°35'N, 30°03'E, 25 April 2009, coll. R.Yu. Dudko, I.I. Lyubechanskij, A.A. Stekolnikov. **Paratypes**: TURKEY: 1 ♂ (ISEA 000.286) Ankara Province, Bala District, Revnam Forests, 1392 m a.s.l., 39°40'N, 32°54'E, 29 May 2009, coll. Yu.M. Marusik; 1 ♂ 1 ♀ (ISEA 000.515) Çankırı Province, Ankara-Çankırı Highway, 689 m a.s.l., 40°23'N, 33°34'E, semidesert, 15 September 2010, coll. Yu.M. Marusik; 4 ♂ (ISEA 000.875) Adıyaman Province, Nemrut Mt., 37°58'N, 38°44'E, 14.05.1997 (V. Bryja); 1 ♂ (LM) Kayseri Province, Nigde, Demirkazık, 37°51'N, 35°05'E, 13 June 1993, coll. C. Felton; 1 ♂ (MNHN 12.840) Amasia [=Amasya], 40°39'N, 35°49'E, date unknown, coll. S.L.; 2 ♂ (NHM) Pass vor Alahan, Karaman ü. Mut [=Mersin Province, Alahan Monastery, nr Mut, 36°47'N, 33°21'E], 8 April 1977, coll. H. Nemenz.

#### Diagnosis.

This species is closely related to *Aelurillus v-insignitus* and other species of *Aelurillus v-insignitus*-group (sensu Azarkina 2006), but differs in the male body coloration, viz. *Aelurillus alboclypeus* sp. n. has a black eye field (Fig. 2) and the abdomen with a few white spots. *Aelurillus v-insignitus* has a V-shaped figure on the eye field and a broad light stripe on dorsum on the abdomen in both the black and grey forms (see Żabka 1997: figs 25, 38), *Aelurillus laniger* Logunov & Marusik, 2000 and *Aelurillus steinmetzi* Metzner, 1999 has a modified V-shaped figure pattern on eye field (see Metzner 1999: fig. 41 a). The clypeus of *Aelurillus alboclypeus* sp. n. is covered with short dense adpressed white hairs (Figs 5, 14) while *Aelurillus v-insignitus* has sparse white hairs (Fig. 18). *Aelurillus guecki* Metzner, 1999 and *Aelurillus laniger* has long shaggy and short yellow-white hairs on clypeus respective and *Aelurillus steinmetzi* has light red hairs. *Aelurillus alboclypeus* sp. n. has dark brown metatarsi and tarsi of leg I and yellow femora, patellae and tibiae (Fig. 15) while *Aelurillus v-insignitus* has yellow femora and brown to dark brown patellae, tibiae, metatarsi and tarsi (Fig. 19). *Aelurillus guecki* has red-brown metatarsi and tarsi of leg I, all legs covered with dark brown hairs. *Aelurillus laniger* has grey femora of leg I ventrally, femora of other legs are brown-grey ventrally. The TA of the embolic division has a small tooth-like process (Figs 7–10) which absent from both forms of *Aelurillus v-insignitus* (Żabka 1997: figs 31, 42) and other *Aelurillus v-insignitus*-group species (see Logunov and Marusik 2000: figs 5–6; Metzner 1999: figs 43 f, h–i). Palpal tibial apophysis both straight and slightly curved dorsally, almost adequate in size (Fig. 4) while palpal tibial apophysis of *Aelurillus laniger* both straight, ventral apophysis slightly longer (Logunov and Marusik 2000: fig. 4), ventral palpal tibial apophysis curved ventrally, small and dorsal palpal tibial apophysis long and straight in *Aelurillus guecki* (Metzner 1999: fig. 40 c), palpal tibial apophysis adequate in size, ventral tibial apophysis slightly curved ventrally and dorsal tibial apophysis straight in *Aelurillus steinmetzi* (Metzner 1999: fig. 41 c), palpal tibial apophysis adequate in size, ventral palpal tibial apophysis bended ventrally and dorsal tibial apophysis slightly curved dorsally in *Aelurillus v-insignitus* (Metzner 1999: fig. 42 c). Females differ from those of *Aelurillus v-insignitus*-group by the poorly visible copulatory openings (Fig. 12).

#### Etymology.

The species is named for its “face coloration”: *Aelurillus alboclypeus* sp. n. has white dense hairs on the clypeus.

#### Description.

Male (holotype (small) and paratype (large) from Demirkazık ): Carapace 2.00–3.10 long, 1.60–2.10 wide, 1.00-1.80 high at PLE. Ocular area 0.95–1.10 long, 1.25–1.60 wide anteriorly and 1.20–1.55 wide posteriorly. Diameter of AME 0.30–0.40. Abdomen 1.90–2.50 long, 1.70–2.10 wide. Cheliceral length 0.65–1.00. Clypeal height 0.25–0.30. Length of leg segments: I 1.3+0.9+0.8+0.5+0.6; II 1.4+0.9+0.8+0.6+0.5; III 2.0+0.9+1.0+1.0+0.8; IV 1.9+0.9+1.2+1.5+0.8. Leg spination: I: Fm d 1–1–5; Pt pr 1; Tb pr 1–1–1, v 1–1–2 ap; Mt pr and rt 1–1, v 2–2 ap. II: Fm d 1–2–5; Pt pr and rt 1; Tb d 1–0–0, pr 1–1–1, v 1–1–2 ap; Mt pr and rt 1–1, v 2–2 ap. III: Fm d 1–3–5; Pt pr and rt 1; Tb d 1–0–0, pr and rt 1–1–1–1, v 1–0–2 ap; Mt d 1–1–0, pr and rt 1–0–2, v 1–1–2 ap. IV: Fm d 1–2–5; Pt pr and rt 1; Tb d 1–0–0, pr and rt 1–1–1–1, v 2–0–2 ap; Mt d 1–1–0, pr 1–1–2, rt 1–0–2, v 1–1–2 ap. Coloration. Carapace dark brown, with black eye field, covered with dark brown to black adpressed scales. Carapace with two thick white stripes dorsally (Fig. 2) and covered with white hairs laterally. Clypeus with short dense white adpressed hairs (Figs 5, 14). Chelicerae dark brown. Abdomen yellow-gray, dorsum black, with an indistinct white longitudinal stripe (Fig. 2) and 5–6 pairs of white indistinct spots in the posterior part of abdomen. Legs yellow-brown. Femur I and II with two yellowish dorsal stripes. Femur I covered prolaterally with dense yellow hairs. Legs III and IV brown. Patella and tibia I and II yellow, covered with short and thin long hairs. Metatarsi and tarsi I and II dark brown (Fig. 15). Palpal femur brown, with a ventral knob, covered dorsally with long white dense hairs. Palpal patella and tibia yellow, with white hairs. Cymbium brown, covered with dark brown hairs. Palpal structure as in Figs 3–4, 7–10.

Female (from Çankırı Prov.): Carapace 2.30 long, 1.30 wide, 1.20 high at PLE. Ocular area 1.00 long, 1.35 wide anteriorly and 1.30 wide posteriorly. Diameter of AME 0.40. Abdomen 2.20 long, 1.40 wide. Cheliceral length 0.70. Clypeal height 0.30. Length of leg segments: I 1.0+0.7+0.7+0.5+0.5; II 1.0+0.7+0.7+0.5+0.45; III 1.7+0.9+0.9+1.0+0.65; IV 1.55+0.7+0.85+1.2+0.7. Leg spination: I: Fm d 1–1–4; Tb pr 1–1, v 1–1–2 ap; Mt pr and rt 1–1, v 2–2 ap. II: Fm d 1–2–4; Tb pr 1–1, v 1–1–2 ap; Mt pr and rt 1–1, v 2–2 ap. III: Fm d 1–2–4; Pt pr and rt 1; Tb d 1–0–0, pr and rt 1–1–1, v 1–0–2 ap; Mt d 1–1–0, pr 1–0–2, rt 1–1–2, v 1–1–2 ap. IV: Fm d 1–1–2; Pt pr and rt 1; Tb d 1–0–0, pr and rt 1–1–1, v 2–0–2 ap; Mt d 1–1–0, pr 1–1–2, rt 1–0–2, v 1–1–2 ap. Coloration. Carapace dark brown with black ocular area, covered with white scales. Sternum dark brown covered with white hairs. Clypeus dark brown covered with white hairs, cheeks dark brown with two strips formatted by dense white hairs. Abdomen grayish-yellow, dorsum dark brown with mixed yellowish-white hair pattern. Book-lungs are grayish-yellow, spinnerets are yellowish-grey. All legs and palps are yellow. Legs with dark brown patches and semi-rings. Structure of spigyne and spermathecae as in Figs 11–13.

**Figure 1.**
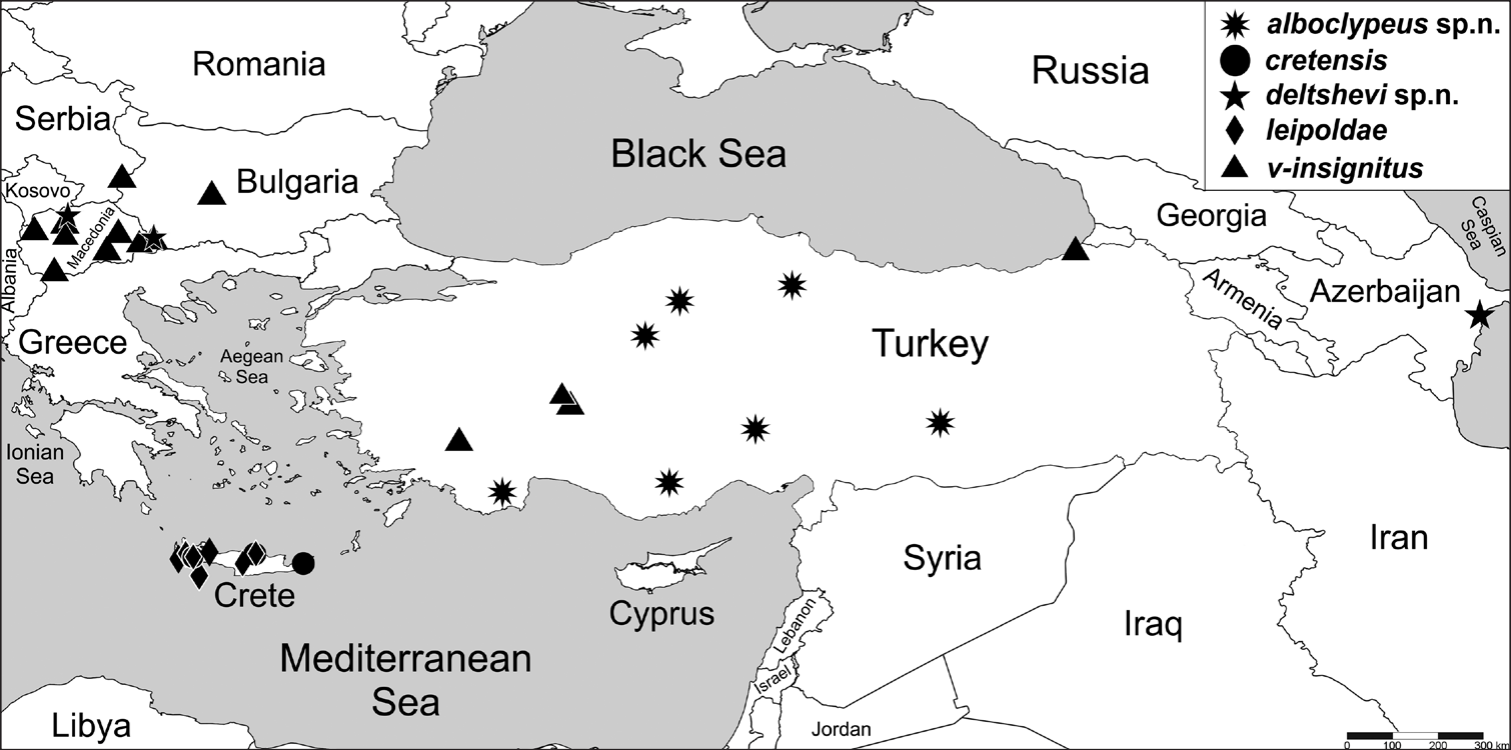
Distributional map of five *Aelurillus* species.

**Figures 2–13.**
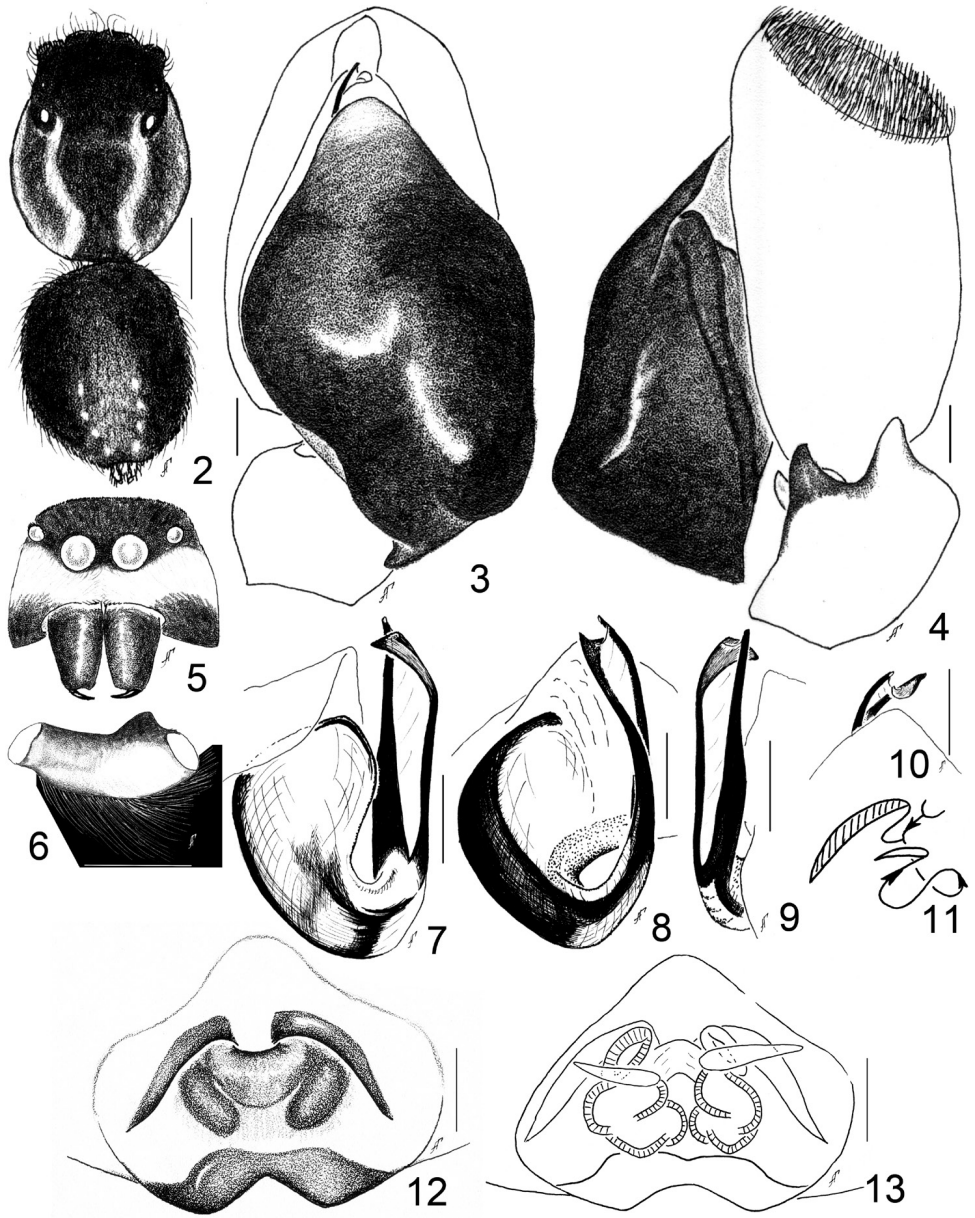
*Aelurillus alboclypeus* sp. n.: **2** male, body pattern **3** left palp, ventral view **4** ditto, retrolateral view **5** male face **6** palpal femur, retrolateral view **7** embolic division, retrolateral view **8** ditto, dorsal view **9** ditto, prolateral view **10** ditto, ventral view **11** diagrammatic course of the insemination ducts **12** epigyne, ventral view **13** spermathecae; dorsal view. Scale bars - 0.1 mm (**3–8, 11–12**), 0.5 mm (**10**); 1 mm (**2**).

**Figures 14–19.**
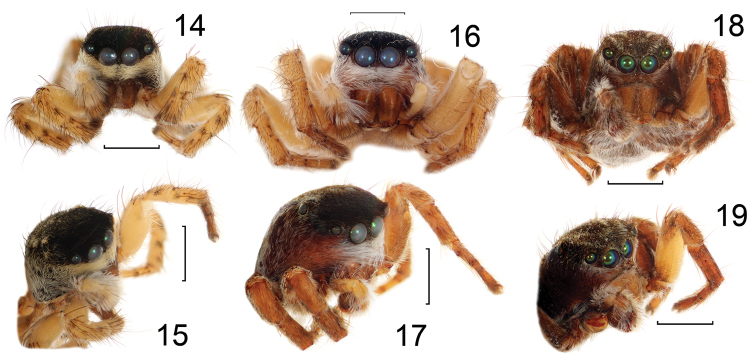
Faces of *Aelurillus alboclypeus* sp. n. (**14–15**), *Aelurillus deltshevi* sp. n. (**16–17**) and *Aelurillus v-insignitus* (Clerck, 1757) (**18–19**). Scale bars - 1 mm.

#### Distribution.

Turkey (Fig. 1).

#### Comments.

First author re-examined *Aelurillus* material from Danişman et al. 2012 and Coşar et al. 2014 (except *Aelurillus luctuosus*) kindly provided by Tarık Danişman in 2013 (*Aelurillus* material is the same in both papers). All *Aelurillus gershomi* belongs to new species, *Aelurillus alboclypeus* sp. n.

### Aelurillus cretensis

Taxon classificationAnimaliaAraneaeSalticidae

Azarkina, 2002

Figs 1 , 32–34

Aelurillus cretensis Azarkina 2002: 251, figs 8–18 (♂♀, re-examined).
Aelurillus steliosi Dobroruka 2002: 8, figs 5–13(allotype ♀, re-examined).
Aelurillus cretensis : Bosmans and Chatzaki 2005: 100 (in part).

#### Type material.

Allotype of *Aelurillus steliosi*: 1 ♀ (MNHN #AR 13335) “GREECE, Crete, Psiloreitis, Kouroutes (Prefectura Irakleio), near Agios Titos church, 1180 m a.s.l., 35°20'N, 25°08'E, 12 June 2001, coll. S. Simaiakis”. Holotype of *Aelurillus cretensis*: ♂ (NHMC) GREECE, Crete, Lefka Ori Mts., 1650 m a.s.l., 35°17'N, 23°54'E, 8 June 1991, coll. P. Lymperakis. Paratypes: GREECE: 1 ♂ (NHMC), 1 ♂ (MMUM) Crete, Lefka Ori Mts., 1650-2100 m a.s.l., 35°17'N, 23°54'E, 16-17 October 1990, coll. P. Lymperakis; 7 ♂ 2 ♀ (ISEA 000.516), 1 ♀ (ISEA 000.517), 2 ♂ 2 ♀ (NHMC), 1 ♀ (MMUM) Crete, Lefka Ori Mts., 1650 m a.s.l., 35°17'N, 23°54'E, 8 June-6 October 1991, coll. P. Lymperakis; 1 ♀ (ISEA 000.711) Crete, Lefka Ori Mts., 2000 m a.s.l., 35°17'N, 23°54'E, 6 August 1992, coll. P. Lymperakis.

#### Other material.

1 ♀ (SNHM) Greece, Crete, Lasithi, mountains S of Sitia, stony, moist beds of stream, under stones and on ground, 35°10'N, 26°06'E, 22 March 1958, coll. H. Kahmann.

#### Distribution.

Only known from Crete, Greece (Fig. 1; Azarkina 2002: fig. 8).

#### Comments.

The male holotype and the female allotype of *Aelurillus steliosi* belong to two different species, *Aelurillus cretensis* (female) and *Aelurillus leipoldae* (male).

Bosmans et al. 2013 erroneously (R. Bosmans, pers. comm.) mentioned *Aelurillus blandus* in reference to *Aelurillus cretensis* (WSC 2015) therefore we excluded this reference from the list.

### Aelurillus deltshevi sp. n.

Taxon classificationAnimaliaAraneaeSalticidae

http://zoobank.org/32281E16-6FEA-4D40-84B9-562CD08673D5

Figs 1 , 16–17 , 20–28

Aelurillus sp. 1: Komnenov 2006: 302
Aelurillus v-insignitus : Lazarov 2005: 151, Tab. 1 (in part).

#### Type material.

**Holotype**: ♂ (IZSB) BULGARIA, Blagoevgrad Province, Strouma Valley, 2 km S of Kamenitsa, 170-240 m a.s.l., 41°38'N, 23°09'E, soil traps, 28 September – 2 February 2002, coll. M. Langourov & S. P. Lazarov. **Paratypes**: MACEDONIA: 1 ♂ (ISEA 000.472) Skopje, Radišani [=Radishani], 42°04'N, 21°27'E, 3 September 1995, coll. M. Komnenov. BULGARIA: 4 ♂ (IZSB) Blagoevgrad Province, Strouma Valley, FM 71, 2 km S of Kamenitsa, 170-240 m a.s.l., 41°37'N, 23°09'E, soil traps, 28 September – 2 February 2002, coll. M. Langourov & S. Lazarov. AZERBAIJAN: 1 ♂ (MMUM) 60 km SW of Baku [=Bakı], Gobustan [=Qobustan], Gobustan Rock Art Cultural Landscape, 40°05'N, 49°24'E, 7.05.1989, coll. P. M. Dunin.

#### Diagnosis.

**Figures 20–28.**
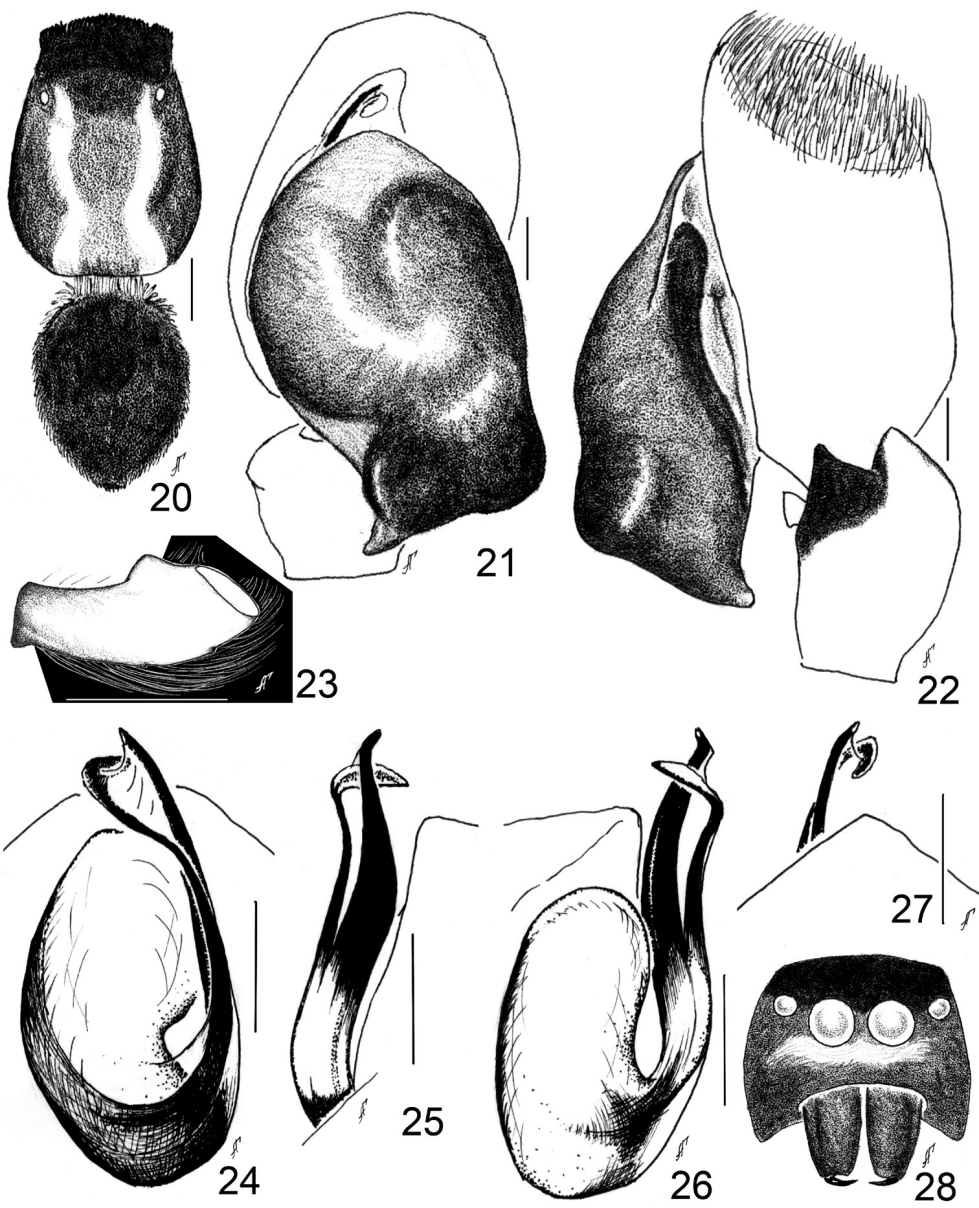
*Aelurillus deltshevi* sp. n.: **20** male, body pattern **21** left palp, ventral view **22** ditto, retrolateral view **23** palpal femur, prolateral view **24** embolic division, ventral view **25** ditto, prolateral view **26** ditto, retrolateral view **27** embolic division, ventral view **28** male face;. Scale bars - 0.1 mm (**21–22, 24–27**), 0.5 mm (**23**); 1 mm (**20**).

*Aelurillus deltshevi* sp. n. belongs to *Aelurillus v-insignitus*-group and is closely related to *Aelurillus alboclypeus* sp. n., *Aelurillus guecki*, *Aelurillus steinmetzi* and *Aelurillus v-insignitus*; it also shares the same colour pattern on the eye field (Fig. 20) with *Aelurillus alboclypeus* sp. n. (Fig. 2) and *Aelurillus guecki* (Metzner 1999: fig. 40 a), but differs from other species of this group which have “V” shape (or its modification) on eye field; the clypeal pattern (a narrow stripe of white hairs under anterior median eyes, Fig. 28), difference in size and shape of the lateral tibial apophysis (Fig. 22, cf. Prószyński 1971: see fig. 18 for *Aelurillus v-insignitus*), and in the structure of the embolic division where the embolus and TA are more curved pro- and retro-laterally, and the apical part of TA is simple, without lateral expansions (Figs 24–27), whereas the apical part of TA of *Aelurillus v-insignitus* is more complicated and laterally expanded, (Metzner 1999: see fig. 43 f), apical part of TA of *Aelurillus alboclypeus* sp. n. with small tooth (Fig. 8), apical part of TA of *Aelurillus guecki* and *Aelurillus steinmetzi* are pointed apically (Metzner 1999: figs. 43 h-i) while apical part of TA of *Aelurillus deltshevi* sp. n. pointed perpendicular to embolus (Fig. 25).

#### Etymology.

This species is named after Prof. Christo Deltshev, the well-known Bulgarian arachnologist.

#### Description.

Male (Paratype, from Bulgaria): Carapace 2.8 long, 2.0 wide, 1.6 high at PLE. Ocular area 1.1 long, 1.55 wide anteriorly and 1.55 wide posteriorly. Diameter of AME 0.45. Abdomen 1.3 long, 1.2 wide. Cheliceral length 1.0. Clypeal height 0.3. Length of leg segments: I 1.4+0.7+0.9+0.65+0.55; II 1.5+0.9+0.9+0.6+0.6; III 1.9+0.9+1.4+1.3+0.65; IV 1.9+0.8+1.3+1.5+0.8. Leg spination: I: Fm d 1–1–5; Pt pr and rt 1; Tb pr 1–2, v 1–1–2 ap; Mt pr and rt 1–1, v 2–2 ap. II: Fm d 1–2–5; Pt pr and rt 1; Tb pr 1–1–1, v 1–1–2 ap; Mt pr and rt 1–1, v 2–2 ap. III: Fm d 1–3–5; Pt pr and rt 1; Tb d 1–0–0, pr and rt 1–1–1–1, v 1–0–2 ap; Mt d 1–1–0, pr and rt 1–0–2, v 1–1–2 ap. IV: Fm d 1–2–5; Pt pr and rt 1; Tb d 1–0–0, pr and rt 1–1–1–1, v 1–0–2 ap; Mt d 1–1–0, pr 1–1–2, rt 1–0–2, v 1–1–2 ap. Coloration: Carapace dark brown, with black eye field, covered with adpressed white scales, more densely on its sides. Carapace with two dorsal longitudinal white stripes. Eye field covered with black shining scales, with no colour pattern (Fig. 20). Clypeus, cheeks and chelicerae brown to dark brown (Fig. 16). Clypeus and cheeks densely covered with white hairs (especially beneath anterior median eyes) (Fig. 28). Hairs around eyes laterally and ventrally white, dorsally black. Abdomen yellow-gray, dorsum dark brown, with thin white hairs. Legs brownish yellow. Metatarsus and tarsus I yellow-brown (Fig. 17). Palps yellow, covered with white hairs, cymbium brown-yellow, covered with brown hairs. Palpal femur with a ventral knob (Fig. 23). Palpal structure as in Figs 21–22, 24–27.

#### Remarks.

*Aelurillus deltshevi* sp. n. was hitherto identified as *Aelurillus v-insignitus*. There are two subspecies of *Aelurillus v-insignitus*, *Aelurillus v-insignitus morulus* (Simon, 1937) from France, and *Aelurillus v-insignitus obsoletus* (Kulczyński in Chyzer and Kulczyński 1891) from Hungary. Simon (1937: p. 1267) commented that in southern France *Aelurillus v-insignitus morulus* would occur together with *Aelurillus v-insignitus*. This species is a local form and can be distinguished from *Aelurillus v-insignitus* by the abdomen and femur coloration (see p. 1227). Kulczyński (1891: p. 30) stated that *Aelurillus v-insignitus* and *Aelurillus v-insignitus obsoletus* were similar in the body colouration, but that of the eye field in *Aelurillus v-insignitus obsoletus* was not adequately visible (“areae huius pictura parum definita”). One of us (GA) tried to find the holotypes of both Simon’s and Kulczyński’s species but failed. It is most likely that they were lost. *Aelurillus deltshevi* sp. n., described here, has the black eye field, without a “V” pattern. According to Kulczyński’s picture (1891: plate 1, figs 4 a–b), the tibial apophysis is typical of *Aelurillus v-insignitus*. The TA in *Aelurillus deltshevi* sp. n. is different as the dorso-lateral branch of the TA in these species is not higher than in *Aelurillus v-insignitus*, and the ventro-lateral branch of the TA is less curved (Figs 24–27). Prószyński (1971) described two forms of *Aelurillus v-insignitus*, “black” and “grey”. Both these forms have visible “V” pattern on the eye field (Prószyński 1971: figs 8-10) and a high dorso-lateral tibial apophysis (Prószyński 1971: figs 13, 16, 18–21). The terminal apophyses of the “black” and “grey” forms are also different from those of *Aelurillus deltshevi* sp. n. (Żabka 1997: figs 30–31, 41–42). However, all of them can easily be separated from *Aelurillus deltshevi* sp. n. by the carapace and clypeal colouration, also by the structure of the embolic division and the shape of the tibial apophysis.

#### Distribution.

Macedonia, Bulgaria and Azerbaijan (Fig. 1).

#### Comments.

*Aelurillus deltshevi* sp. n. occurs in Macedonia and Bulgaria at the elevations below 500 m a.s.l., while *Aelurillus v-insignitus* has been recorded from the elevations above 500 m a.s.l..

### Aelurillus leipoldae

Taxon classificationAnimaliaAraneaeSalticidae

(Metzner, 1999)

Figs 1 , 29–31

Asianellus leipoldae Metzner 1999: 72, figs 37 a–i (♂, SMNK, re-examined).
Aelurillus leipoldae : Azarkina 2002: 253, figs 31–42; Logunov and Chatzaki 2003: 96.
Aelurillus steliosi Dobroruka 2002: 8, figs 5–13 (♂, re-examined) **syn. n.**
Aelurillus cretensis : Bosmans and Chatzaki 2005: 100 (in part); Bosmans et al. 2013: 110 (in part).

#### Type material.

Holotype of *Aelurillus steliosi*: ♂ (MNHN #AR 13334) “GREECE, Crete, Skalani (Pref. Irakleio), 230 m a.s.l., 35°17'N, 25°11'E, 21 May 2001, coll. S. Simaiakis”. Holotype of *Asianellus leipoldae* ♂ (SMNK, 2177) “GREECE, Kreta, Paleohóra, Küstengebirge” [=Crete, Palaiochora, Coastal Ranges, 35°13'N, 23°40'E], 9.01.1993 (D. Leiopold). Paratype of *Asianellus leipoldae* 1 ♂ (PCHM) “Kreta [=Crete], Chania, 35°18'N, 23°48'E, 4.09.1974 (A. Senglet).

#### Other material.

GREECE: 10 ♂ 2 ♀ (ISEA 001.4045, 001.4047, 001.4058) Crete, Chania, Lefka Ori Mts., 800 and 1650 m a.s.l., 35°17'N, 23°54'E, 23 November 1990, 6 July–6 November 1991, coll. P. Lymperakis; 2 ♀ (ISEA 001.4057) Gavdos Island, Chania, Lavrakas sand-dunes, *Juniperus* forest, 34°52'N, 24°04'E, 24 July–8 November 1997, coll. K. Paragamian; 1 ♂ (LM) Crete, September 2002, coll. S.L. Felton; 1 ♀ (SNHM) Crete, Chania, N of Lake Curna [=Kournas], N slope, 100 m from the coast, *Luminacea, Salvia*, 0-15 m a.s.l., 35°20'N, 24°16'E, 16 April 1958, coll. H. Kahmann; 1 ♀ (SNHM) Crete, Heraklion, 2 km SE of Zaros, NE slope, flat hill, sandy, *Phrygana, Cirsium, Cystus*, under stones, 35°07'N, 24°55'E, 7 April 1958, coll. H. Kahmann.

#### Remarks.

**Figures 29–34.**
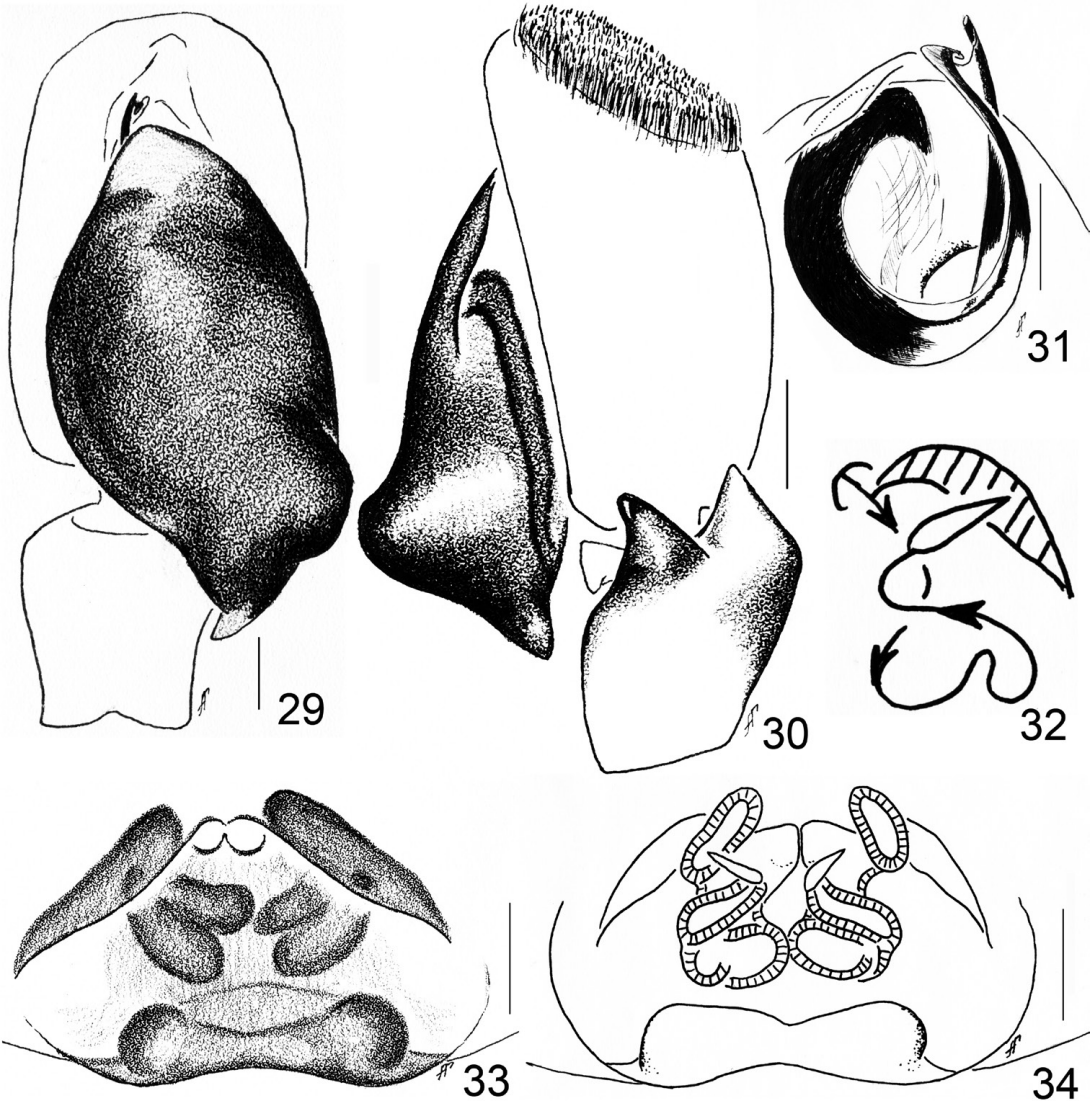
Male of *Aelurillus leipoldae* (holotype of *Aelurillus steliosi*) and female of *Aelurillus cretensis* (allotype of *Aelurillus steliosi*): **29** left palp, ventral view **30** ditto, retrolateral view **31** embolic division, dorsal view **32** diagrammatic course of the insemination ducts **33** epigyne, ventral view **34** spermathecae, dorsal view. Scale bars - 0.1 mm (**29–31, 33–34**).

The holotype of *Aelurillus steliosi* is conspecific with that of *Aelurillus leipoldae*. Both specimens examined (the male holotypes of *Aelurillus leipoldae* and *Aelurillus steliosi*) have the same body coloration and structure of the palpus and the embolic division (Figs 29–31 and see Azarkina 2002: figs 31–38, 41; Dobroruka 2002: figs 6–10). Therefore, it is safe to conclude that the name *Aelurillus steliosi* Dobroruka, 2002 is a junior synonym of *Aelurillus leipoldae* (Metzner, 1999), contrary to Bosmans et al. (2013) (see also comment under *Aelurillus cretensis*) who synonymized *Aelurillus steliosi* with *Aelurillus cretensis* Azarkina, 2002.

#### Distribution.

Only known from Crete, Greece (Fig. 1; Metzner 1999: map 29; Azarkina 2002: fig. 43).

## Supplementary Material

XML Treatment for Aelurillus alboclypeus

XML Treatment for Aelurillus cretensis

XML Treatment for Aelurillus deltshevi

XML Treatment for Aelurillus leipoldae

## Appendix

### *Aelurillus v-insignitus* (Clerck, 1757)

**Material** (the studied material was partly published by Komnenov 2002, 2003, 2006 and Logunov 2015). MACEDONIA: 1 ♂ (PCMK) Šar Planina [=Shar] Mt., 1320 m a.s.l., 41°48.455'N, 20°47.862'E, pitfall traps, 19 July 1998 (M. Komnenov); 3 ♀ (PCMK) Pelister [=Baba] Mt., 1200-1500 m a.s.l., 41°01.760'N, 21°13.369'E, July 2001 (M. Komnenov); 2 ♂ 1 ♀ (PCMK) Demir Kapija, 550 m a.s.l., 41°20.843'N, 22°18.334'E, 21 May 2005 (M. Komnenov); 1 ♂ (PCMK) Jakupica Mt., 2000 m a.s.l, 41°40.670'N, 21°24.245'E, 11 July 1999 (M. Komnenov); 1 ♂ (PCMK) Plačkovica [=Plachkovica] Mt., 1700 m a.s.l, 41°45'N, 22°28'E, 8-20 July 2002 (M. Komnenov); 1 ♂ (PCMK) Vodno Mt., Skopje, 41°57'N, 21°23'E, 26 April 2003 (M. Komnenov); 1 ♂ (PCMK) Vodno Mt., Skopje, 41°57.972'N, 21°23.890'E, 5 May 2002 (M. Komnenov); 1 ♂ (PCMK) Ogražden [=Ograzhden] Mt., 41°33.719'N, 22°49.440'E, 14 July 2000 (M. Komnenov). BULGARIA: 1 ♂ (IZWP) Zelenigrad near Tran, 42°50'N, 22°33'E, 2 May 1966 (V. Beškov, W. Staręga); 1 ♂ 2 ♀ (IZSB) FM 71, Soil traps, Pirin N.P., Struma [=Strouma] River valley (South), 2 km S of Kamenica [=Kamenitsa], 1700-2400 m a.s.l., 41°37'N, 23°09'E, 5 April – 9 May 2002 (M. Langourov & S. P. Lazarov); 1 ♂ (IZSB) Săštinska Sredna Gora [=Sredna Gora] Mt. Range, Strelča [=Strelcha], 700 m a.s.l., 42°30'N, 24°19'E, 9 May 1998 (S. P. Lazarov). TURKEY: 1 ♂ (ISEA) Artvın Province, Hopa, 41°23'N, 41°25'E, 17 May 1997 (V. Bryja).
